# Acetyl CoA Carboxylase 2 Is Dispensable for CD8^+^ T Cell Responses

**DOI:** 10.1371/journal.pone.0137776

**Published:** 2015-09-14

**Authors:** Jang Eun Lee, Matthew C. Walsh, Kyle L. Hoehn, David E. James, E. John Wherry, Yongwon Choi

**Affiliations:** 1 Department of Pathology and Laboratory Medicine, Perelman School of Medicine, University of Pennsylvania, Philadelphia, Pennsylvania, 19104, United States of America; 2 Department of Microbiology, Perelman School of Medicine, University of Pennsylvania, Philadelphia, Pennsylvania, 19104, United States of America; 3 Institute for Immunology, Perelman School of Medicine, University of Pennsylvania, Philadelphia, Pennsylvania, 19104, United States of America; 4 Department of Pharmacology, University of Virginia Health System, Charlottesville, Virginia, 22908, United States of America; 5 Charles Perkins Centre Research and Education Hub, University of Sydney, Sydney, New South Wales, Australia; Maisonneuve-Rosemont Hospital, CANADA

## Abstract

Differentiation of T cells is closely associated with dynamic changes in nutrient and energy metabolism. However, the extent to which specific metabolic pathways and molecular components are determinative of CD8^+^ T cell fate remains unclear. It has been previously established in various tissues that acetyl CoA carboxylase 2 (ACC2) regulates fatty acid oxidation (FAO) by inhibiting carnitine palmitoyltransferase 1 (CPT1), a rate-limiting enzyme of FAO in mitochondria. Here, we explore the cell-intrinsic role of ACC2 in T cell immunity in response to infections. We report here that ACC2 deficiency results in a marginal increase of cellular FAO in CD8^+^ T cells, but does not appear to influence antigen-specific effector and memory CD8^+^ T cell responses during infection with listeria or lymphocytic choriomeningitis virus. These results suggest that ACC2 is dispensable for CD8^+^ T cell responses.

## Introduction

CD8^+^ T cells are the primary population responsible for protective immunity to infection with intracellular pathogens and cancer. Upon antigen (Ag) encounter, CD8^+^ T cells undergo an expansion phase and an effector phase, followed by a contraction phase, as a result of the orchestration of cell-extrinsic and-intrinsic factors. Subsequently, only a small subset of cells survives to differentiate into memory cells that ensure faster and more efficient immune protection against later infection. During differentiation, phenotypic and functional changes of T cells are evident, and metabolic signatures at each differentiation stage or within each specific subset of T cells have been increasingly appreciated [1–5]. Proliferating effector T cells switch to glycolysis, while quiescent naïve or memory T cells predominantly utilize oxidative phosphorylation (OXPHOS) by using glucose, amino acids, and fatty acids as carbon sources. Also, a recent study suggests that the superior capacity of memory T cells for fatty acid oxidation (FAO) supports their long-term survival [1]. However, despite recent advances in characterizing metabolic reprogramming and energy-yielding processes during T cell differentiation, it remains unclear whether or how these metabolic characteristics work as deterministic factors in T cell differentiation.

Acetyl-CoA carboxylase enzymes, ACC1 or ACC2, catalyze the conversion of acetyl CoA to malonyl CoA, and their activity is regulated by AMP-activated protein kinase (AMPK)-mediated phosphorylation. ACC1 localizes primarily to the cytosol to produce malonyl CoA, which serves as a carbon donor for fatty acid (FA) synthase-mediated long-chain FA synthesis. ACC2 is anchored along the mitochondrial surface where its synthesis of malonyl CoA works as an allosteric inhibitor of carnitine palmitoyl transferase 1 (CPT1), which regulates transport of long chain fatty acids into the mitochondria for subsequent FAO. The structure and enzymatic function of both ACC proteins have long been characterized at a sub-molecular level, and are conserved across many species [6, 7]. ACC2 knockout mice exhibit increased FAO and reduced fat accumulation in their adipose tissue [8]. These mice are protected against high fat and high carbohydrate diet-induced obesity. Additionally, tissue-specific deletions of ACC2 in heart [9] or skeletal muscle [10] exhibit enhanced FAO in the targeted tissues. Together, these studies suggest ACC2 plays a critical role in regulating FAO and cellular catabolism. However, the cell-intrinsic role of ACC2 in T cell homeostasis, differentiation, and function has not been studied.

Previously, we and others have shown that pharmacological agents that enhance FAO by modulating the AMPK/mammalian target of rapamycin (mTOR) pathway, such as metformin and rapamycin, promote differentiation of memory CD8^+^ T cells and increase vaccine efficacy [11, 12]. These results suggest that there could be a direct cause and effect relationship between FAO and CD8^+^ T cell fate decision processes. Here, we genetically targeted ACC2 specifically in T cells in order to eliminate possible non-specific or off-target effects of pharmacological agents, and directly examined the contribution of ACC2 to CD8^+^ T cell immune responses.

## Materials and Methods

### Mice and infections

*ACC2* ^*f/f*^ mice (from Dr. David E. James, University of Sydney, Australia) on C57BL/6 background were crossed to *Cd4*-Cre mice. *ACC2* ^*f/+*^ *Cd4-*Cre^+^ or *ACC2* ^*f/f*^ *Cd4-*Cre^-^ littermates (WT) were used as controls in all experiments. Total 40 WT or 42 ACC2ΔT mice were infected with either recombinant attenuated *L*. *monocytogenes* (LmOVA) or lymphocytic choriomeningitis virus (LCMV) Armstrong in order to look at primary effector, memory, and secondary effector CD8^+^ T cell differentiation. For primary infections, mice were infected intravenously (i.v.) with 1×10^6^ CFU of LmOVA [13] or intraperitoneally (i.p.) with 2 × 10^5^ PFU LCMV Armstrong. For rechallenge infections, mice were infected i.v. with 1× 10^7^ CFU of LmOVA or 1 × 10^6^ PFU LCMV Cl13. Mice were kept a maximum of eight to nine weeks after infection. All mice were provided access to food and water ad libitum, and were visually monitored daily. We did not observe any signs of suffering or death caused by infection with the given dose. Euthanasia at the completion of experiments was carried out by carbon dioxide asphyxiation.

### Ethics Statement

All mice were housed according to the policies of the Institutional Animal Care and Use Committee of the University of Pennsylvania and all studies were performed in accordance with the recommendations in the Guide for the Care and Use of Laboratory Animals of the National Institutes of Health. The experiments performed with mice in this study were approved by the Institutional Animal Care and Use Committee (IACUC protocol # 803071), University of Pennsylvania (Animal Welfare Assurance Number A3079-01).

### Cell isolation, purification, and flow cytometry

Lymphocytes were prepared and stained for flow cytometric analysis as described elsewhere [14]. Absolute cell numbers were calculated based on the percentage of specific population from the total cells acquired as determined by flow cytometric analysis. Anti-KLRG-1 and CD127 antibodies were purchased from Biolegend. For intracellular cytokine staining, single-cell suspensions from spleens were cultured at 37°C in complete RPMI 1640 supplemented with Golgiplug (BD Biosciences) in the presence of SIINFEKL, H-2D^b^/GP33, or H-2D^b^/NP396 peptide for 5 hrs. After surface staining with anti-CD8 antibody (Ab), cell suspensions were fixed and permeabilized by Cytofix/Cytoperm solution (BD Biosciences) followed by staining with anti-IFN-γ (XMG1.2) or TNF-α (MP6-XT22) Abs. Stained cells were collected with an LSRII (BD Biosciences) and analyzed with FlowJo software (TreeStar). For naïve cell purification, mononuclear cells from spleen and lymph nodes were enriched for CD8^+^ T cells using magnetic separation beads (Miltenyi Biotec). After MACS enrichment, cells were stained with anti-CD44, CD62L, CD25, CD8, and CD4 Abs to further sort out naïve CD8^+^ T cells (CD44^low^CD62L^high^CD25^neg^) by FACSAria (BD Biosciences).

### Fatty acid oxidation assay

Naïve CD8^+^ T cells were incubated with [9,10-^3^H]-palmitic acid (Perkin Elmer) complexed to fatty acid free BSA (Sigma) for 11 hours in low-glucose Dulbecco’s modified Eagle medium media (1000 mg/L glucose, Invitrogen) containing 10% FBS. ^3^H_2_O was quantitated by running supernatants through a Dowex 1x8-200 anion exchange column (Sigma-Aldrich), and β-oxidation was calculated as the difference between oxidation counts in the presence or absence of etomoxir, an irreversible inhibitor of CPT1. [3, 11].

### Statistical analysis

All data are presented as mean ± standard deviation. The mixed-effect model or the two-tailed unpaired Student's *t*-test was used for comparison of the two groups using customized routines written in the statistical programming language R (version 2.15.0). In all cases, p < 0.05 was considered statistically significant.

## Results

### Deletion of ACC2 enhances cellular FAO in CD8^+^ T cells

**Fig 1:**
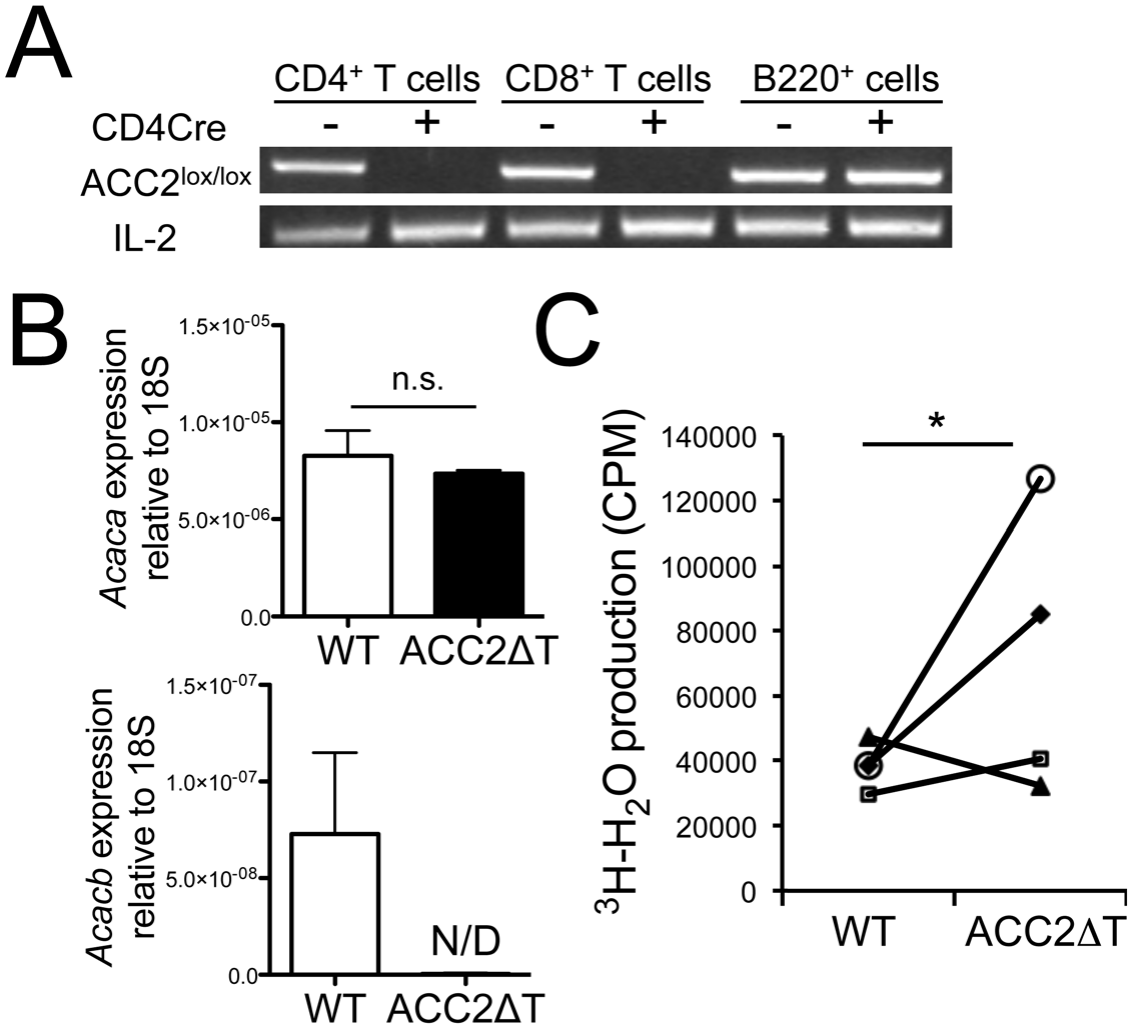
Effects of ACC2 deletion on cellular FAO in T cells. (A) T cell-specific deletion of the *ACC2* gene; PCR analysis confirms ACC2 deletion in genomic DNA in FACS-purified CD4^+^, CD8^+^, B220^+^, CD11b^+^ cells from *ACC2* ^*f/f*^ (WT) and *ACC2* ^*f/f*^ *Cd4-Cre* (ACC2ΔT) mice. IL-2 was an internal control. (B) Real-time quantitative PCR analysis of *ACC1* or *ACC2* (exon 12) expression from WT or ACC2ΔT CD8^+^ T cells. Results are presented relative to 18S. (C) FACS purified naïve CD8^+^ T cells from WT and ACC2ΔT mice were incubated with ^3^H-palmitate for 11 hours, and supernatants assayed for production of ^3^H_2_O as detailed in Methods. Analysis was performed in triplicate or quadruplicate. Each dot represents independent experiments, and the graph depicts paired analyses of WT and ACC2ΔT cells performed four times independently (Mixed-effect model, p = 0.0032).

To investigate the role of ACC2 in CD8^+^ T cell immune responses, we crossed mice in which exon 12 of the gene encoding ACC2, *Acacb*, is flanked by *lox*P sites to *CD4*-Cre transgenic mice to induce T cell–specific deletion. Deletion of targeted exon 12, encoding a critical region of the biotin carboxylase motif [10], in peripheral T cells was confirmed by PCR analysis of genomic DNA (Fig 1A). *Acacb* mRNA was not detected in ACC2ΔT CD8^+^ T cells, while mRNA for *Acaca*, which encodes the cytosolic ACC, appeared unchanged (Fig 1B). In order to evaluate whether ACC2 regulates cellular FAO in T cells, we measured in vitro oxidation of ^3^H-palmitate to ^3^H_2_O with CD8^+^ T cells. No differences were observed in mitochondrial mass between WT and ACC2ΔT CD8^+^ T cells (data not shown), and interestingly, ACC2ΔT CD8^+^ T cells exhibited only slightly altered FAO compared to WT CD8^+^ T cells in naïve conditions (Fig 1C), suggesting only marginal contributions by ACC2 to FAO regulation in CD8^+^ T cells.

### Loss of ACC2 does not affect T cell homeostasis in the periphery

**Fig 2:**
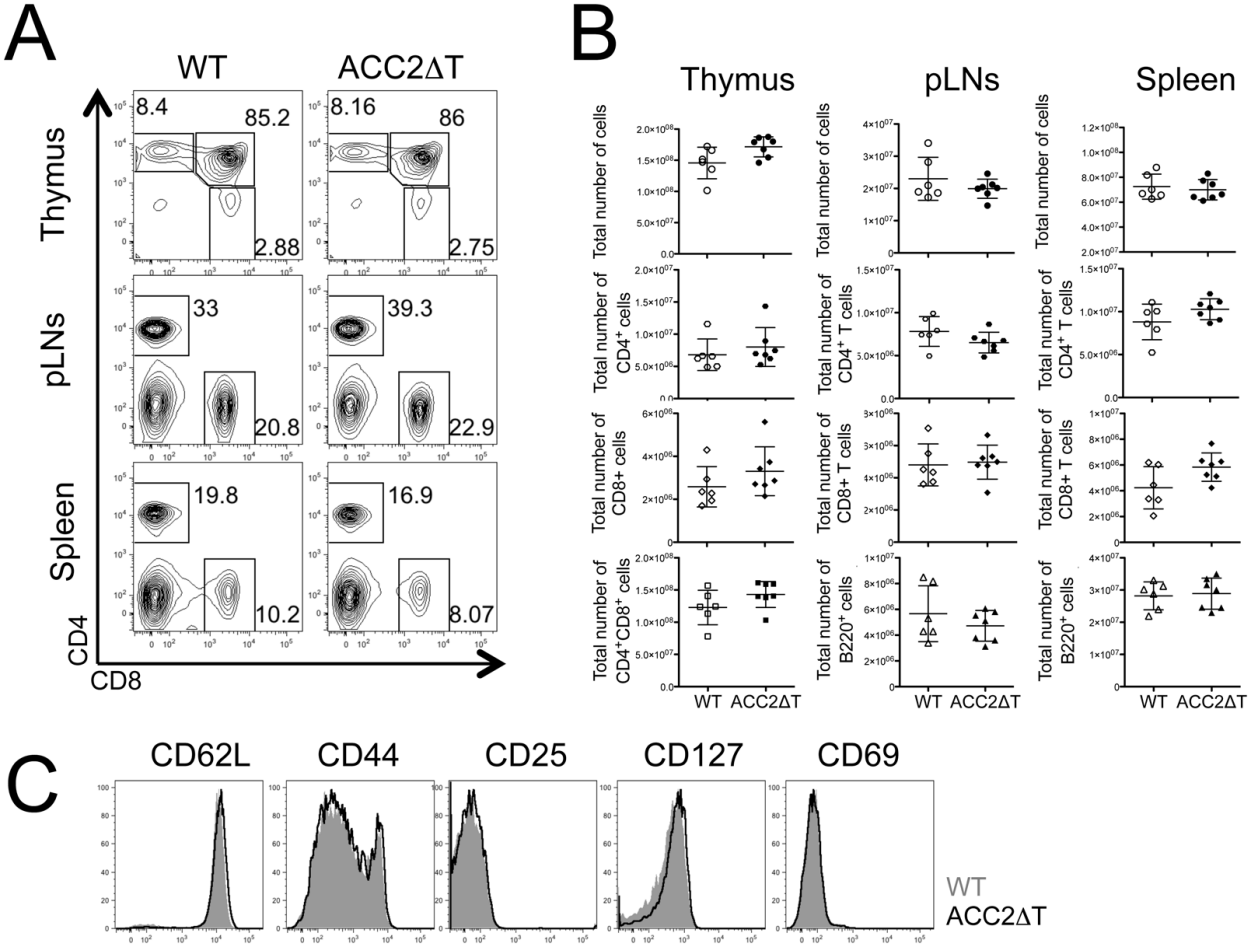
Loss of ACC2 does not affect T cell homeostasis in the periphery. (A) Frequency of CD4^+^ and CD8^+^ T cells in the thymus, peripheral lymph nodes (pLNs), and spleen from naive ACC2ΔT and WT littermate mice (7–12 weeks old). Shown are representative dot plots from at least three independent experiments. (B) Numbers of isolated cells in the spleen and thymus from ACC2ΔT mice and WT littermates (means ± standard deviation) (C) Expression of various surface markers in splenic CD8^+^ T cells from WT (grayed area) and ACC2ΔT (black line) mice.

Next we examined the effects of ACC2 deletion on peripheral T cell homeostasis by analyzing the frequency and numbers of T cells in thymus, spleen, and peripheral lymph nodes (pLN) isolated from ACC2ΔT and WT littermate mice. ACC2ΔT mice did not show any gross differences in frequencies (Fig 2A), numbers (Fig 2B), or activation status (Fig 2C) of CD8^+^ T cells in all tissues examined, suggesting that lack of ACC2 *per se* does not affect T cell homeostasis. We previously reported that T cell-specific deletion of ACC1 impairs T cell homeostasis in the periphery [13]. Consistent with studies showing distinct roles for ACC1 and ACC2 in fatty acid metabolism [15], our results also demonstrate differential requirements for ACC1 and ACC2 in CD8^+^ T cell homeostasis.

### ACC2 expression is dispensable for CD8^+^ T cell primary, memory, and recall responses during LmOVA or LCMV infection

**Fig 3:**
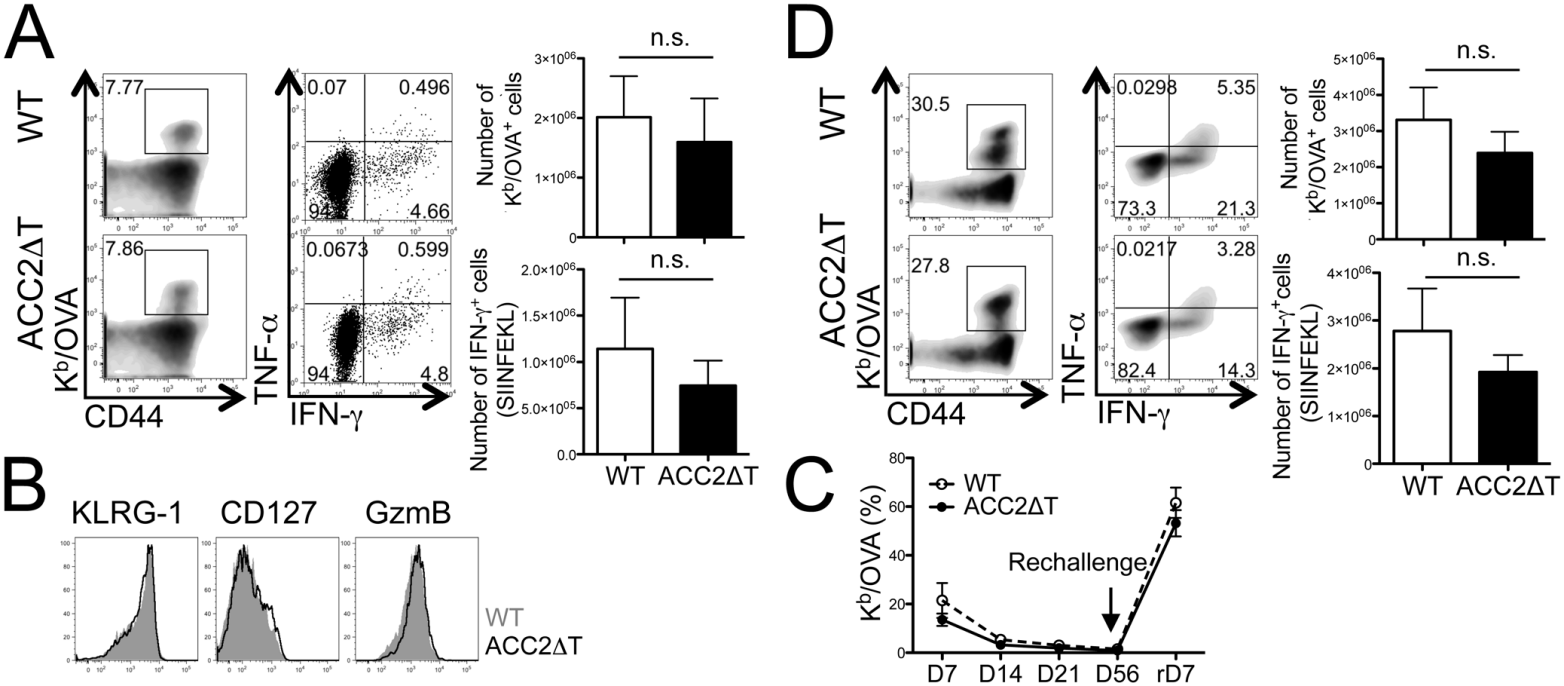
ACC2 is dispensable for Ag-specific CD8^+^ T cell responses during LmOVA infection. (A) WT or ACC2ΔT mice were infected with LmOVA. Seven days post-infection, single cells were prepared from spleens and stained for various surface markers and cytokines. Dot plots show frequency of K^b^/OVA tetramer positive cells (numbers indicate percent of total CD8^+^ T cells) or IFN-γ- and TNF-α−producing WT and ACC2ΔT CD8^+^ T cells. Graph represents number of K^b^/OVA tetramer positive cells or IFN-γ-producing WT and ACC2ΔT CD8^+^ T cells (means ± standard deviation). Data are representative of three independent experiments (WT = 12, ACC2ΔT = 12). (B) The expression of surface markers and granzyme B was determined in WT (grayed area) and ACC2ΔT (black line) cells seven days post infection. (C) Frequency of K^b^/OVA tetramer positive cells from the blood at the indicated time points (D) Frequency (dot plot) or number (graph) of K^b^/OVA tetramer positive cells (numbers indicate percent of total CD8^+^ T cells) or IFN-γ- and TNF-α−producing WT and ACC2ΔT CD8^+^ T cells after secondary infection. Mice were re-challenged 56 days post primary infection. Shown here is representative of three independent experiments (WT = 10, ACC2ΔT = 12).

To investigate the role of ACC2 in differentiation of CD8^+^ T cells, we infected mice with two different pathogens, LmOVA or LCMV Armstrong, and examined Ag-specific CD8^+^ T cell responses. Both LmOVA and LCMV infections result in robust expansion of CD8^+^ T cells accompanied by effector and memory CD8^+^ T cell differentiation, yet they elicit distinctive T cell-extrinsic and-intrinsic factors [16]. After infection with LmOVA, both WT and ACC2ΔT mice exhibited equivalent expansion of OVA-specific CD8^+^ T cells as well as efficient expression of effector molecules, such as IFN-γ, TNF-α, and granzyme B (Fig 3A and 3B). No difference was found in the expression of surface markers, such as KLRG-1 and CD127 (Fig 3B). Next we examined whether ACC2 plays a role in the contraction or differentiation/maintenance of memory T cells. Serial bleeds showed comparable kinetics of the frequency of OVA-specific CD8^+^ T cells in the blood from both WT and ACC2ΔT mice over time (Fig 3C). Upon re-challenge, ACC2ΔT mice showed normal secondary expansion and effector functions as WT mice did (Fig 3D), demonstrating that deletion of ACC2 does not influence the development of anti-bacterial CD8^+^ T cell immune responses.

**Fig 4:**
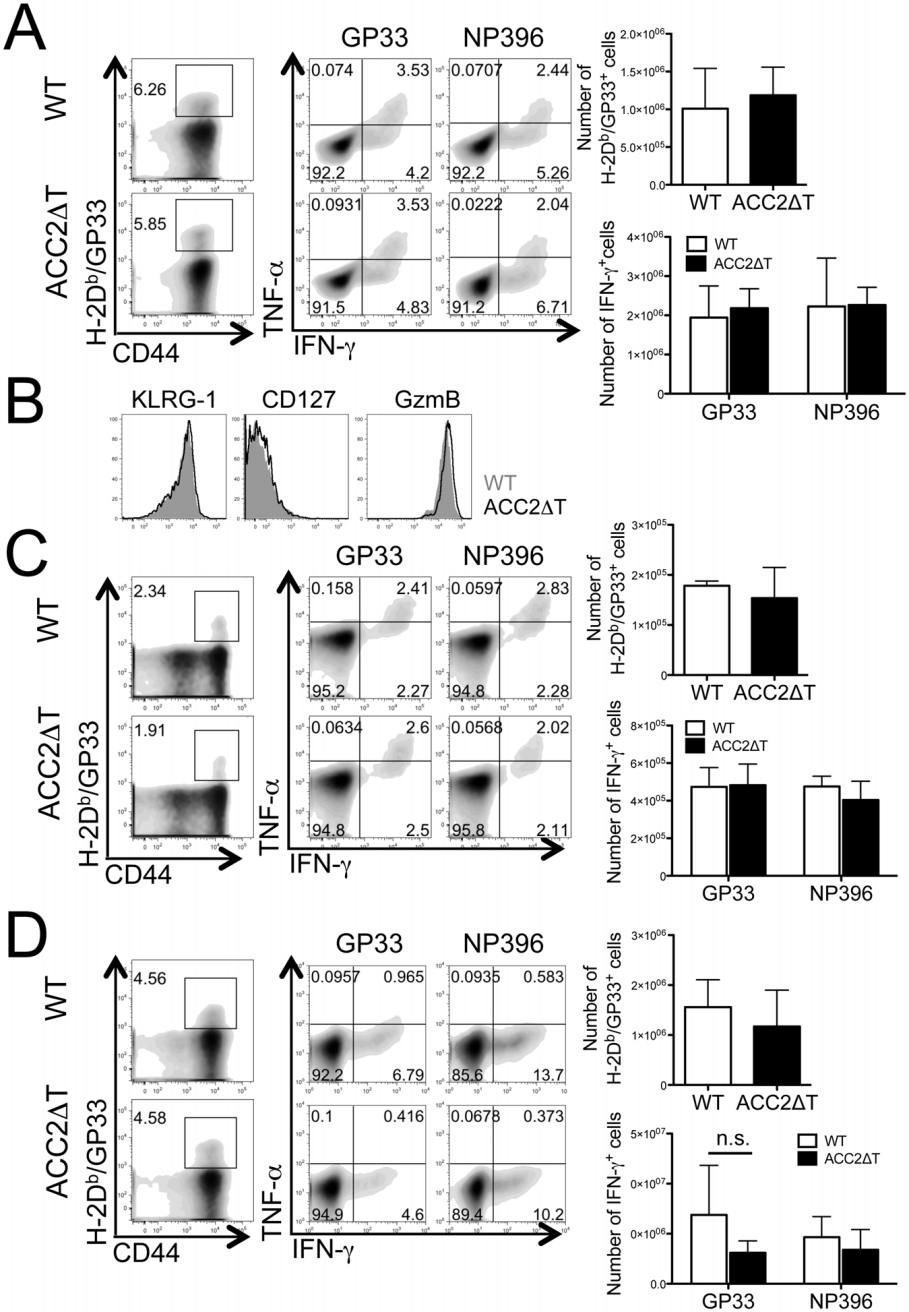
ACC2 is not essential for Ag-specific CD8^+^ T cell responses during LCMV infection. (A) WT or ACC2ΔT mice were infected with LCMV Armstrong. Dot plots show frequency of H-2D^b^/GP33 tetramer positive cells (numbers indicate percent of total CD8^+^ T cells) or IFN-γ- and TNF-α−producing WT and ACC2ΔT CD8^+^ T cells seven days post infection. For detection of IFN-γ- and TNF-α−producing CD8^+^ T cells, cells were re-stimulated with GP33 or NP396 peptides as indicated. Graph represents number of H-2D^b^/GP33 tetramer positive cells or IFN-γ-producing WT and ACC2ΔT CD8^+^ T cells (means ± standard deviation). Shown here is a representative of two independent experiments (WT = 7, ACC2ΔT = 7). (B) The expression of surface markers and granzyme B was determined in WT (grayed area) and ACC2ΔT (black line) H-2D^b^/GP33 tetramer positive cells seven days post infection. (C) Frequency (dot plots) or number (graphs) of H-2D^b^/GP33 tetramer positive cells or GP33- or NP396-specific IFN-γ- and TNF-α−producing WT and ACC2ΔT CD8^+^ T cells 56 days post infection. Shown here is a representative of two independent experiments (WT = 6, ACC2ΔT = 6). (D) 56 days post primary infection, mice were rechallenged with Cl13 and analyzed five days later for frequency (dot plots) or number (graphs) of H-2D^b^/GP33 tetramer positive cells or GP33- or NP396-specific IFN-γ- and TNF-α−producing WT and ACC2ΔT CD8^+^ T cells. Data are from two independent experiments (WT = 5, ACC2ΔT = 5).

Anti-viral CD8^+^ T cell responses were measured after LCMV Armstrong infection. WT and ACC2ΔT mice showed equivalent primary LCMV-specific CD8^+^ T cell responses (Fig 4A) as evidenced by the similar number of GP33-tetramer positive or GP33- or NP396-specific IFN-γ-producing CD8^+^ T cells upon peptide re-stimulation. No difference was found in the expression of surface markers, such as KLRG-1 and CD127, or the effector molecule, granzyme B (Fig 4B). Subsequently, ACC2ΔT mice exhibited normal development of memory (Fig 4C) and recall responses (Fig 4D) after LCMV Cl13 rechallenge. Together, these data indicate that ACC2 is not essential for primary expansion or memory CD8^+^ T cell differentiation in response to LCMV infection.

## Discussion

In this study, we demonstrated that acetyl CoA carboxylase 2 (ACC2) is dispensable for CD8^+^ T cell homeostasis, activation, and differentiation. This result demonstrates significant differences in the roles of ACC1 and ACC2 in controlling CD8^+^ T cell immunity, emphasizing the importance of further dissecting the localization, expression patterns, and functions of each isoform in cellular metabolism, which is often driven by multiple isoforms that may perform redundant or compensatory functions.

Recent studies have suggested that memory CD8^+^ T cell differentiation, survival, or function is closely correlated with FAO [1, 3, 11, 17]. After contraction, CD8^+^ T cells heavily relied on OXPHOS for energy yielding, and FA-fueled OXPHOS (hereafter referred as FAO) appeared to be associated with memory CD8^+^ T cell function. Additionally, enhancing FAO using retroviral overexpression of CPT1a, a rate-limiting enzyme in FAO, in CD8^+^ T cells increased the number of Ag-specific cells when analyzed 14 days post-Lm infection [1]. Along with our previous result showing the positive effect of metformin or rapamycin treatment in memory CD8^+^ T cell differentiation [11], other current data [1, 12] seemingly suggest that tipping the energy-yielding process toward FAO could be a viable means of controlling CD8^+^ T cell differentiation. Therefore, it is important and necessary to understand the role of fatty acid-associated metabolic components in CD8^+^ T cell immunity. However, our data concerning T cell-specific deletion of ACC2 may suggest that FAO regulation of memory CD8^+^ T cell differentiation occurs in a multifactorial rather than binary manner, as we did not detect clear constitutive enhancement of FAO in ACC2-deficient CD8^+^ T cells. Regardless, it is clear that genetic ablation of ACC2 activity—whether that activity does or does not involve the cellular FAO pathway *per se*—does not play an essential role in regulating CD8^+^ T cell homeostasis and differentiation from naïve to effector phases, or from effector to memory phases. Therefore, with respect to potential direct roles for FAO in T cell fate decisions, more sophisticated analyses and/or additional gene targets must be employed in order to make more conclusive findings. It will also be important in the future to consider temporal changes in cellular availability of metabolites or other substrates including enzymes in bioenergetic pathways, which could be key factors in regulating effector function [18, 19] or transition from effector to memory CD8^+^ T cells. Additionally, it could be important to consider relative changes of other reciprocal bioenergenic pathways, for instance glycolysis, in relation to FAO. It is also possible that observed effects on FAO profiles might be a secondary to fluctuation of other metabolic pathways that direct cellular function or fate. For example, Sukumar M et al. showed that enforcing glycolysis by overexpression of Pgam1 suppressed memory CD8^+^ T cell differentiation, while inhibiting glycolysis using 2-deoxy glucose, glucose analogue, showed the opposite [20]. In this study, cells with both reduced extra cellular acidification rate (ECAR; proxy measurement of glycolysis) and oxygen consumption rate (OCR; proxy measurement of OXPHOS) were likely to become memory CD8^+^ T cells, suggesting that what matters could be glucose starvation signaling and/or AMPK triggered by enforced cellular quiescence rather than an increase of OXPHOS as a compensatory mechanism in response to blocking glycolysis.

In this study, we showed that ACC2 is dispensable for CD8^+^ T cell homeostasis and immune function, and surprisingly, may not play a biologically significant T cell-intrinsic role in regulating FAO. However, it remains that elucidating the roles of cellular metabolism in T cell function and differentiation have important implications for developing cell-specific immunomodulation strategies or vaccine design. Therefore, further studies into characterization of the elements of cellular metabolism as biomarkers will be important.
